# Rethinking Food Anticipatory Activity in the Activity-Based Anorexia Rat Model

**DOI:** 10.1038/srep03929

**Published:** 2014-01-29

**Authors:** Hemmings Wu, Kris van Kuyck, Tim Tambuyzer, Laura Luyten, Jean-Marie Aerts, Bart Nuttin

**Affiliations:** 1 Research Group Experimental Neurosurgery and Neuroanatomy, KU Leuven, Leuven, Belgium; 2 M3-BIORES: Measure, Model & Manage Bioresponses, Department of Biosystems, KU Leuven, Leuven, Belgium; 3 Psychology of Learning and Experimental Psychopathology, Faculty of Psychology and Educational Sciences, KU Leuven, Leuven, Belgium; 4 Department of Neurosurgery, University Hospitals Leuven, Leuven, Belgium

## Abstract

When a rat is on a limited fixed-time food schedule with full access to a running wheel (activity-based anorexia model, ABA), its activity level will increase hours prior to the feeding period. This activity, called food-anticipatory activity (FAA), is a hypothesized parallel to the hyperactivity symptom in human anorexia nervosa. To investigate in depth the characteristics of FAA, we retrospectively analyzed the level of FAA and activities during other periods in ABA rats. To our surprise, rats with the most body weight loss have the lowest level of FAA, which contradicts the previously established link between FAA and the severity of ABA symptoms. On the contrary, our study shows that postprandial activities are more directly related to weight loss. We conclude that FAA alone may not be sufficient to reflect model severity, and activities during other periods may be of potential value in studies using ABA model.

Routtenberg and Kuznesof first described the relationship between an increase in running activity and a decrease in food intake in rats in 1967^1^. They discovered that when rats were on a restricted feeding schedule (1 hour per day in their experiment) and had free access to a running wheel, their food intake was significantly lower than in control rats, which were on the same feeding schedule but without access to a running wheel. This discrepancy between increased running activity and decreased food intake caused substantial body weight loss, and if rats were not removed from the experimental setup timely, they would eventually die of starvation. This model, later named the activity-based anorexia (ABA) model, is one of the most widely used animal models for the study of anorexia nervosa (AN)^2^.

AN is a serious psychiatric disorder most prevalent in adolescent and young females^3^. It is multifactorial, the etiology behind is complicated to say the least, and it includes various clinical symptoms, but two of the most noticeable physiological manifestations are self-induced pathological body weight loss and excessive exercising, and the ABA model exhibits both of these features^2^,^4^. Unlike other psychiatric disorders in which specific psychiatric evaluations are the main measures of disease progress (e.g. the Yale–Brown Obsessive Compulsive Scale for obsessive-compulsive disorder), the Body Mass Index (body mass (kg) divided by the square of height (m)) is viewed as the main clinical indicator of disease progress and treatment efficacy^5^,^6^. Correspondingly, the rat's body weight is the key measure in the ABA model, but besides body weight, the running wheel activity (RWA, or hyperactivity, quantified in number of wheel rotations) is another important measure to assess in this animal model of AN.

The excessive running activity causes calorie depletion in rats, and the logical idea behind treating rats in the ABA model is: if one could reduce running activity, rats could conserve energy better, which may lead to body weight increase and higher survival rate. Moreover, animal and clinical studies have indicated that the hyperactivity in anorectic patients is more than a method to lose weight; it may be a core element and a psychological drive involved in the evolution of the disease^2^. The hyperactivity in the ABA model peaks 2–3 hours before the scheduled feeding^7^. This specific peak in running activity prior to the scheduled feeding, called food-anticipatory activity (FAA), is an important feature of the ABA model. The FAA peak increases over time as rats are re-exposed to scheduled feeding, and it is generally argued that it is an indicator of disease progress and treatment effect besides body weight and survival rate: a decrease in FAA is often interpreted as a sign related to an improvement of the anorectic state, though not always correlated with body weight increase and higher survival rate^8^,^9^,^10^,^11^,^12^.

After a decade of experience with the ABA model, we have observed considerable inter-subject variability. Using the exact same ABA protocol, different rats exhibit different levels of susceptibility to the model. In other words, after 10 consecutive days of whole-day access to a running wheel and scheduled food restriction, the weight loss varies in a relatively wide range. Given this variability and the variability in FAA - a complex circadian behavior - we speculated that characteristics of FAA (e.g. level and pattern) may be correlated with the extent of body weight loss.

Based on a general consensus in previous studies (decrease in FAA indicates positive effect of treatment)^8^,^9^,^10^,^11^,^12^, our main hypothesis is as follows: the higher the amount of FAA a rat demonstrates, the more likely it will lose substantial body weight in the ABA model. We further hypothesize that certain characteristics of FAA could be used as a prognostic indicator, which could predict the percentage of body weight loss in the ABA model. In this study, we investigated the characteristics of FAA and activities during other periods, and their correlations with body weight in 56 ABA rats.

## Results

Based upon their final percentage body weight (last day of ABA procedure), rats were categorized into 3 groups: NS: 88.80 ± 0.75% (mean ± standard error of the mean), n = 13; MS: 81.07 ± 0.55%, n = 26; HS: 66.91 ± 0.87%, n = 17.

**Figure 1:**
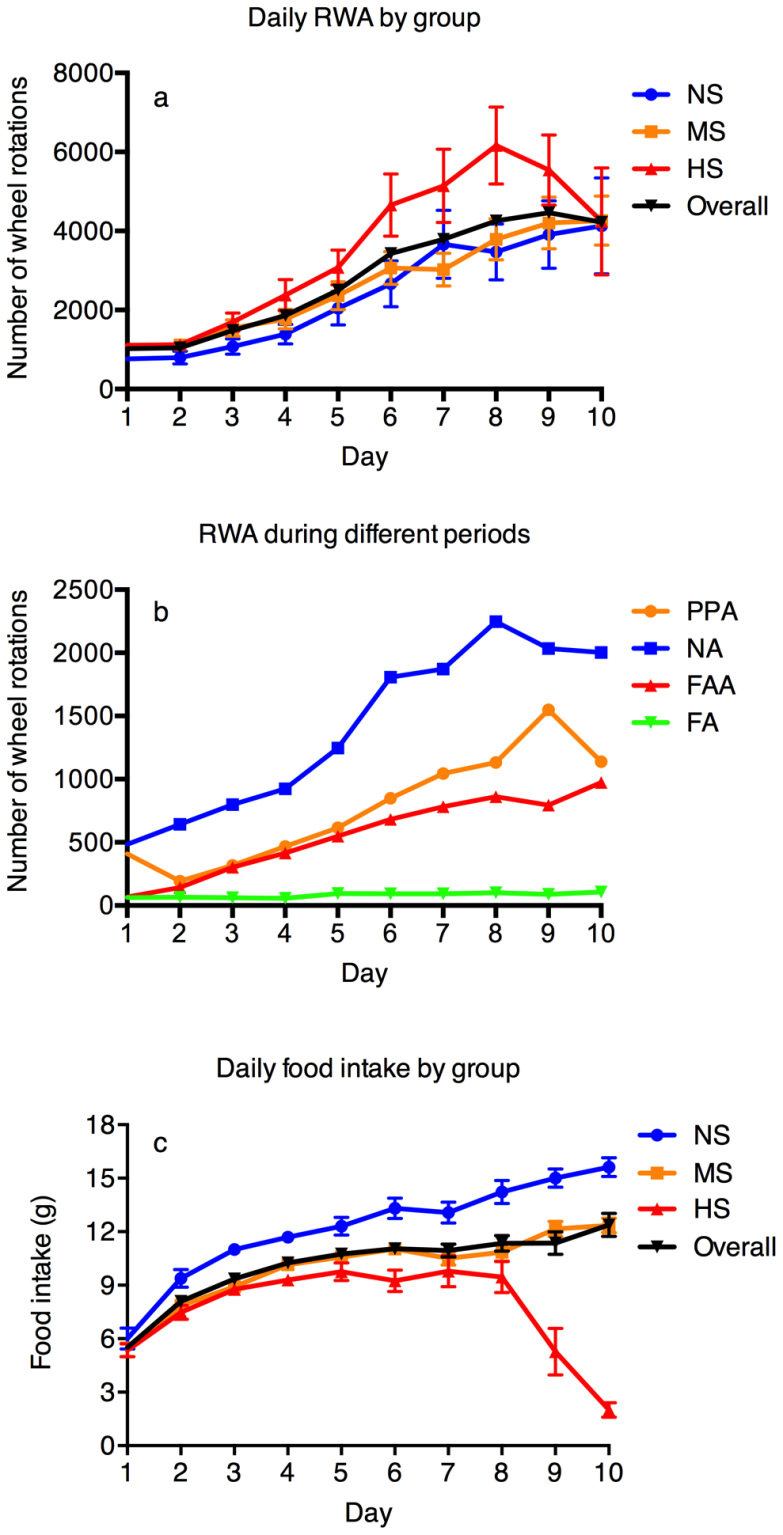
Evolution of RWA of different groups (1a) and during different periods of time (1b), and evolution of food intake (1c). (1a): Increases in daily RWA were observed in all three groups of rats (most noticeable in the HS group). Daily RWA started to decline in the HS group after day 8, probably related to the increasing weakness of rats nearing the 70% criterion, and early dropouts of the more hyperactive rats. (1b): RWA of all rats during different periods of time, showing a trend of increase in FAA, PPA and NA over time. (1c): Daily food intake was highest in the NS group and lowest in the HS group in general, which confirmed insufficient food intake as a factor of body weight loss in this model. The average daily food intake in HS group (8.06 ± 0.27 g) was significantly lower than in the MS (9.94 ± 0.23 g) and NS groups (12.16 ± 0.32 g). RWA: running wheel activity, NS: non-susceptible group, MS: moderately-susceptible group, HS: highly-susceptible group, PPA: postprandial activity, NA: nocturnal activity, FAA: food anticipatory activity, FA: feeding activity.

The evolution of daily RWA is plotted in figure 1A. Rats suffering from the highest percentage weight loss (HS group) manifested the highest level of RWA, which resonates the hypothesis that hyperactivity is playing a major role in pathological weight loss in this model. There was a decrease in daily RWA after day 8 in the HS group, probably related to the increasing weakness of rats nearing the 70% criterion and early dropouts of the more hyperactive rats. On average, the RWA increased by four-fold after 10 days in the ABA model (from 1025 on day 1 (n = 56) to 4221 on day 10 (n = 45), U test, p < 0.01). Figure 1B shows the change in RWA during different periods. Despite food restriction, there was a clear trend of increase in FAA, PPA, and NA over time (day 1 compared to day 10, U test, p < 0.01 for all parameters), in alignment with previous findings. Figure 1C indicates the change in food intake over time. The average daily food intake in the HS group (8.06 ± 0.27 g) was significantly lower than in the MS (9.94 ± 0.23 g) and NS groups (12.16 ± 0.32 g) (ANOVA, p < 0.01, post-hoc: HS:MS, p < 0.01, HS:NS, p < 0.01, MS:NS, p < 0.01).

**Figure 2:**
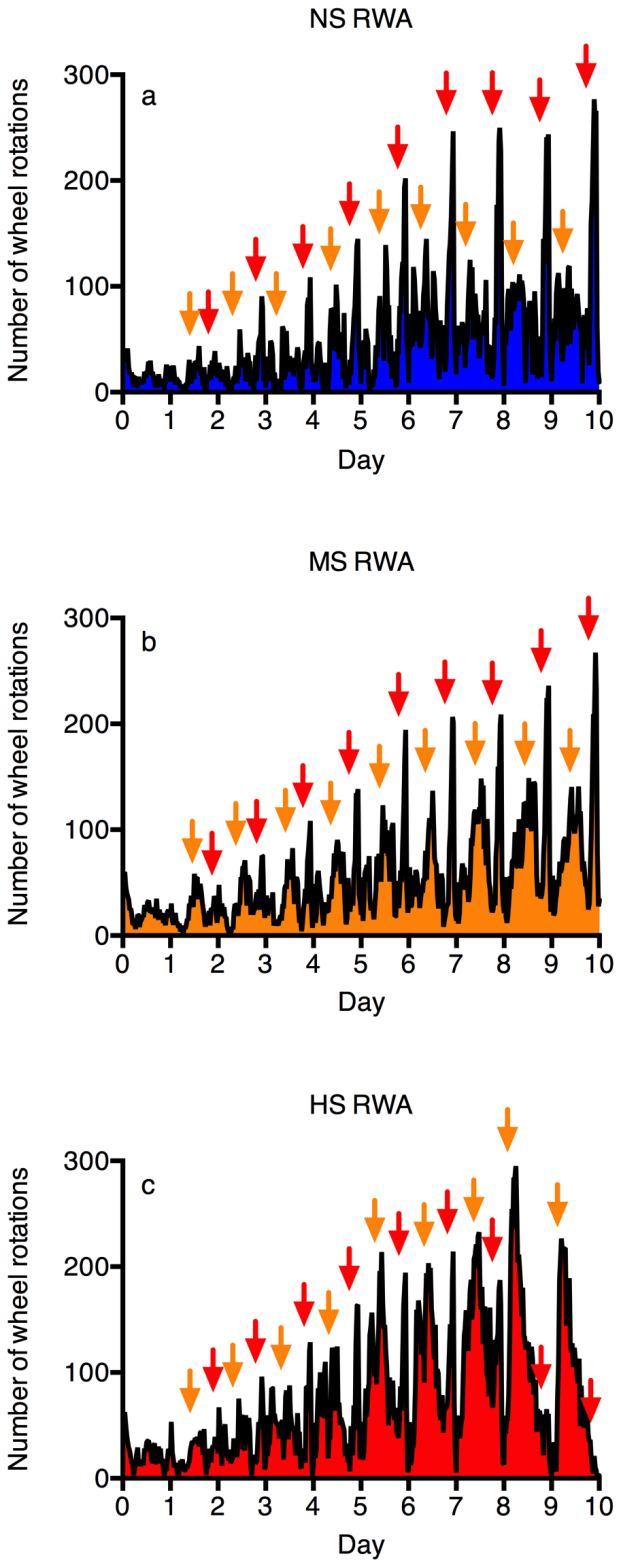
Evolution of number of wheel rotations over time in the NS (a), MS (b), and HS (c) groups. 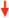
 indicates peaks in RWA, which correspond to FAA; 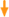 indicates peaks in RWA, which span from PPA and NA. The level of PPA-NA peaks was the lowest in the NS group (a), becoming more distinct in the MS group (b), and reached its maximum height and width in the HS group, surpassing the FAA peaks starting on day 5 (c). RWA: running wheel activity, NS: non-susceptible group, MS: moderately-susceptible group, HS: highly-susceptible group, PPA: postprandial activity, NA: nocturnal activity, FAA: food anticipatory activity.

Figure 2 shows the average 10-day RWA evolution of NS, MS, and HS groups. FAA peaks (red arrows) formed distinctively in all three groups of rats at similar levels. Secondary peaks (orange arrows) were present between FAA, spanning from PPA to NA, but they were the lowest and the narrowest in the NS group by visual inspection, becoming higher and wider in the MS group, and reached maximal height and width in the HS group (peak surpassing level of FAA starting on day 5 in the HS group).

**Figure 3:**
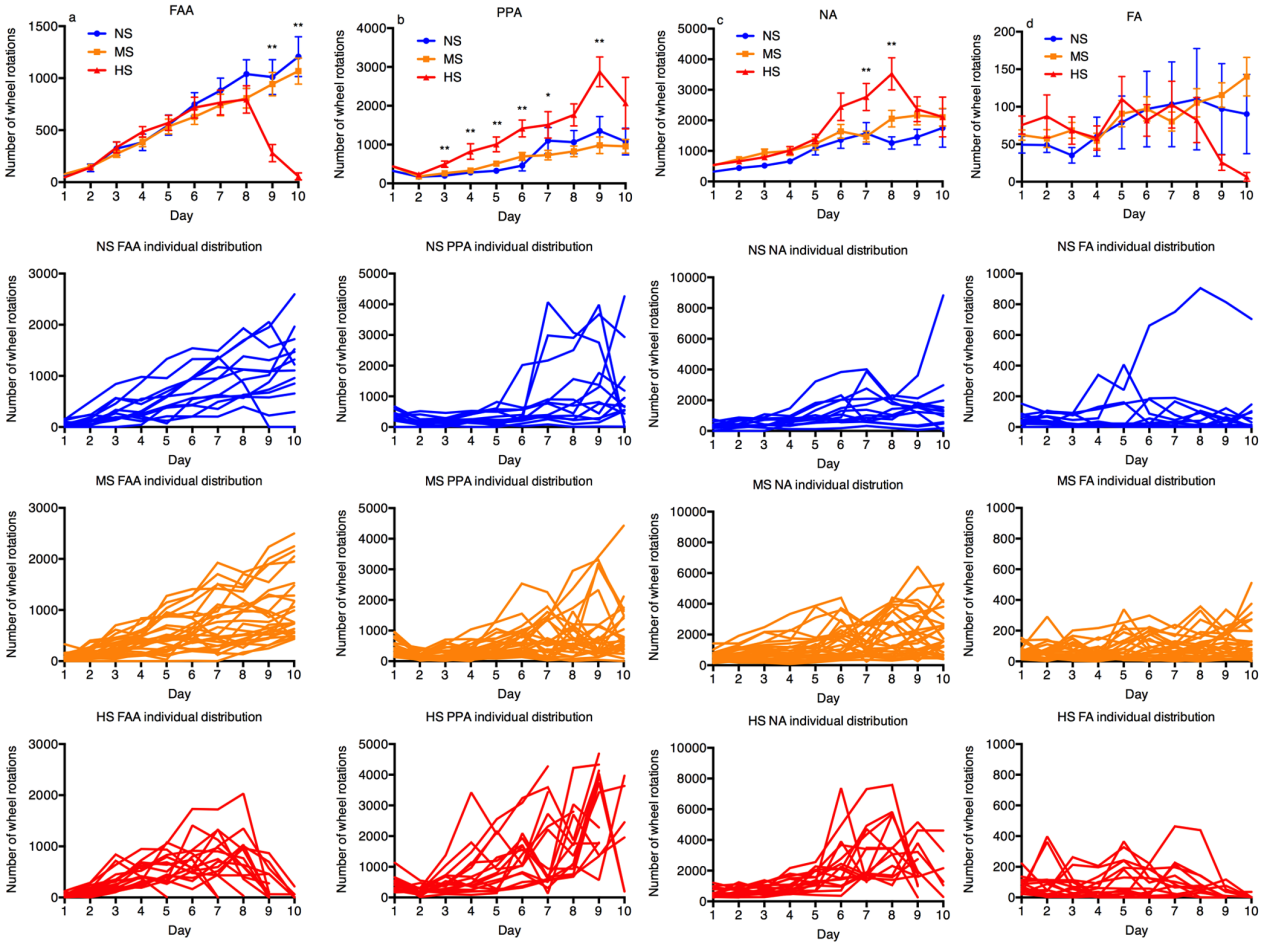
Comparison of FAA, PPA, NA and FA between NS, MS, and HS groups (mean ± standard error of the mean in the top graph and individual raw data of each group (spaghetti graph) in the bottom three graphs). (a): Increases in FAA were shown in all three groups in the first 8 days of the ABA procedure (difference of sample mean not significant), but FAA was significantly lower in the HS group than the rest on day 9 and 10. (b): PPA in the HS group was significantly higher than the rest on day 3, 4, 5, 6, 8 and 9. (c): NA was significantly higher in the HS group on day 7 and day 8. (d): Change of FA over time was less clear, though it was significantly lower in the HS group on day 9 (than the MS group) and day 10 (than both the MS and the HS groups.). NS: non-susceptible group, MS: moderately-susceptible group, HS: highly-susceptible group, PPA: postprandial activity, NA: nocturnal activity, FAA: food anticipatory activity; *: p < 0.05, **: p < 0.01 (analysis of variance, Tukey's post-hoc tests).

Comparison of RWA of different groups during different periods is made in figure 3. FAA, PPA and NA in all three groups were increasing consistently during the first 8 days. FAA in the HS group was not significantly different from FAA in the MS and the NS group (ANOVA). However, PPA was significantly higher in the HS group on day 3, 4, 5, 6, 8 and 9 compared to the two other groups (ANOVA: day 3, 5, 6, 9: p < 0.01, post-hoc, HS:MS and HS:NS, p < 0.01; day 4: p < 0.01, post-hoc, HS:MS, p < 0.01, HS:NS, p < 0.05; day 8: p < 0.05; post-hoc, HS:MS, p < 0.01). NA was also significantly higher in the HS group on day 7 and 8 (ANOVA: day 7, p < 0.01; post-hoc, HS:NS, p < 0.05, HS:MS, p < 0.01, day 8, p < 0.01; post-hoc, HS:NS, p < 0.01, HS:MS, p = 0.01). FA was significantly lower in the HS group on day 9 and 10 (ANOVA, day 9, p < 0.05, post-hoc, HS:MS, p < 0.01; day 10, p < 0.05, post-hoc, HS: MS and HS:NS, p < 0.01). Individual RWA of each rat from each group was plotted to illustrate the distribution of raw data, and despite deviations, its general impression further confirms our results based on group averages.

**Figure 4:**
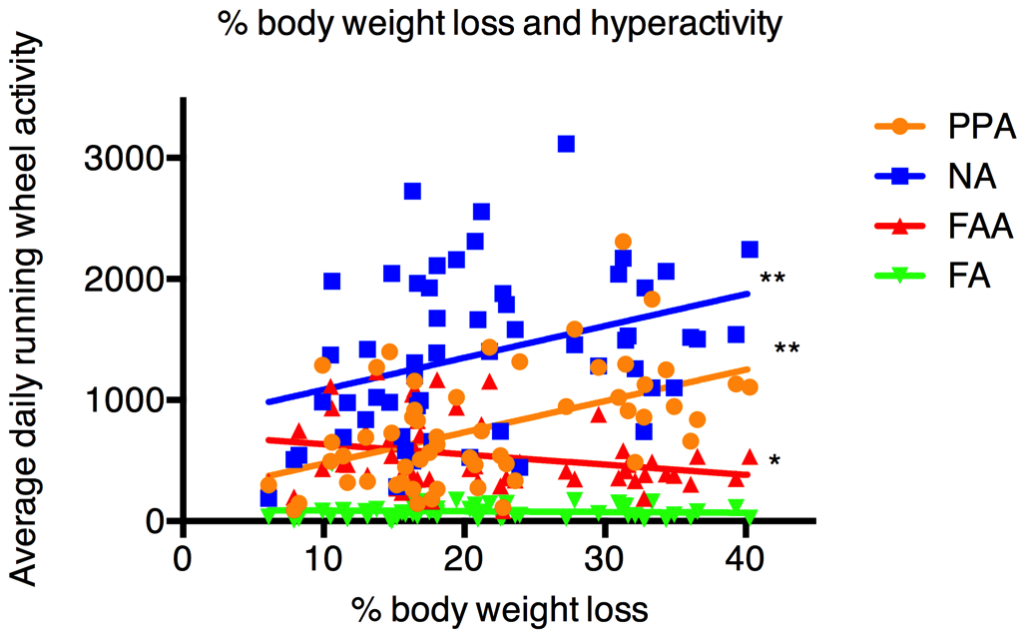
Total percentage body weight loss in relation to average daily running wheel activity during different periods (individual data with linear regression line). Pearson correlation of FAA, PPA, NA, and FA were −0.27 (p < 0.05), 0.49 (p < 0.001), 0.35 (p < 0.01), and −0.07 (p > 0.1), respectively. PPA: postprandial activity, NA: nocturnal activity, FAA: food anticipatory activity, FA: feeding activity; *: p < 0.05, **: p < 0.01.

The daily averages of running wheel activity during different periods in relation to total percentage body weight loss were plotted in figure 4. Pearson correlation of FAA, PPA, NA, and FA were −0.27 (p < 0.05), 0.49 (p < 0.001), 0.35 (p < 0.01), and −0.07 (p > 0.1), respectively.

**Figure 5:**
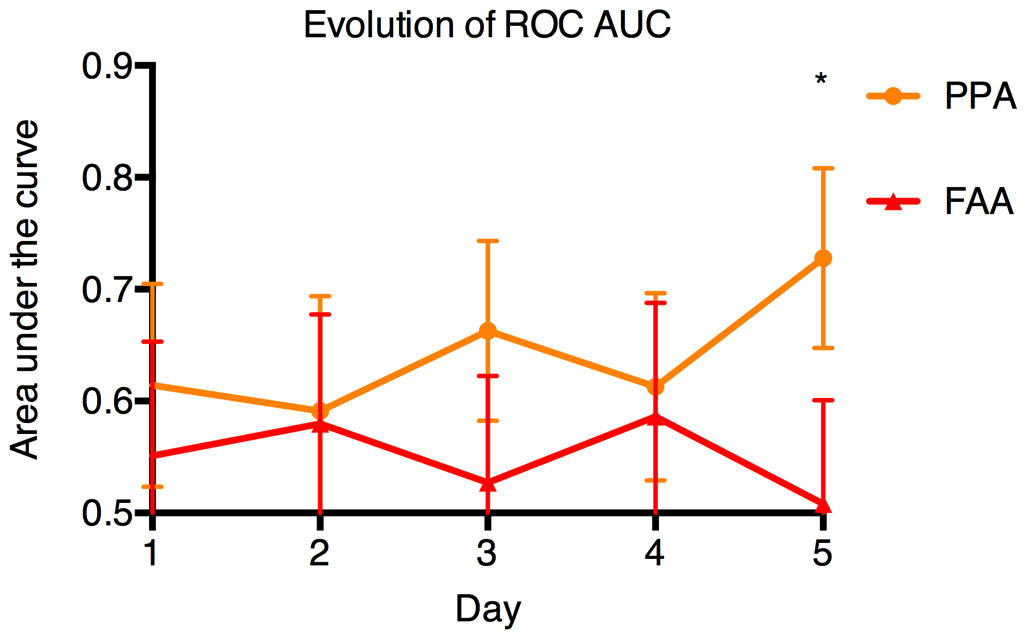
Change in ROC AUC of PPA and FAA over time. The PPA ROC AUC were higher than the FAA ROC AUC in the first five days in the ABA model (significant on day 5, * = p < 0.01). ROC AUC: the area under the receiver operating characteristic curve, FAA: food anticipatory activity, PPA: postprandial activity.

The changes in ROC AUC values of PPA and FAA are shown in figure 5. ROC AUC of PPA was significantly higher (p < 0.01) than that of FAA on day 5, indicating better predicting value in the PPA in terms of distinguishing the responders (MS and HS) from the non-responders (NS) group. ROC AUC of FA and NA were not significantly higher than that of FAA, and were not shown in this figure.

**Figure 6:**
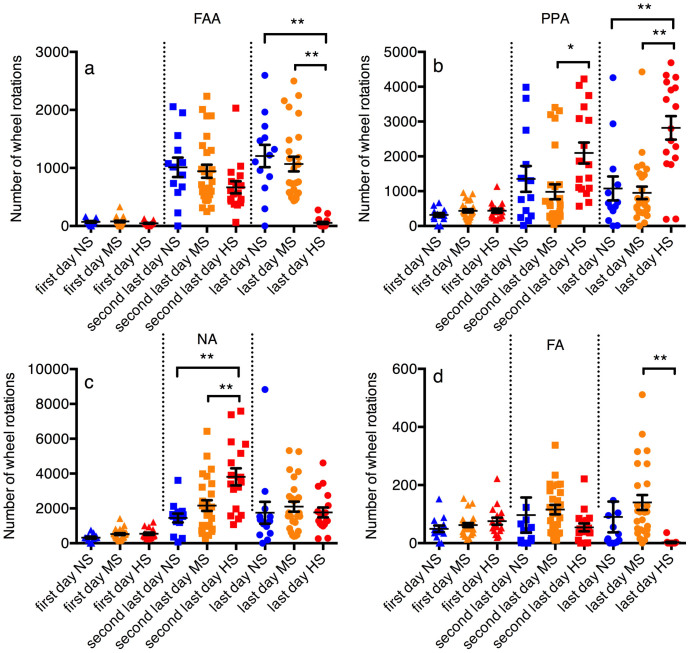
Comparison of FAA, PPA, NA and FA between groups on different days (in terms of before dropout). (a): FAA was lower in the HS group than the rest on the second last and the last day in the ABA cage/model (significant on the last day (p < 0.01)). (b): PPA was higher in the HS group than the rest on the second last and the last day in the ABA cage (second last day: HS significantly higher than MS (p < 0.05) but not NS; last day: HS significantly higher than both MS and NS (p < 0.01). (c): NA was higher in the HS group on the second last day than the NS group and the MS group (p < 0.01), and was not the highest in the HS group on the last day (sample mean difference insignificant). (d): FA was the lowest in the HS group on the last day in the ABA cage (significantly lower than the MS group, p < 0.01). For more detailed graphical representation of the overall data, two data points were not shown (but included in the statistical analysis) in figure 5D (one in second last day of NS and one in last day of NS, valued 814 and 704, respectively). NS: non-susceptible group, MS: moderately-susceptible group, HS: highly-susceptible group, PPA: postprandial activity, NA: nocturnal activity, FAA: food anticipatory activity, FA: feeding activity; *: p < 0.05, **: p < 0.01 (analysis of variance, Tukey's post-hoc tests).

Since early dropouts may induce bias in the HS group, we made a direct between-group comparison of FAA, PPA, NA and FA based on the rats data on the first day, the second last day, and the last day of the ABA procedure (for instance, if a rat was dropped out on day 8, day 8 would be the last day, and day 7 would be the second last day for this rat) (figure 6). The results were similar to the findings in figure 3. The mean FAA values were the lowest in the HS group compared to NS and MS, on the second last day (not significant, ANOVA, p > 0.1) and the last day in the ABA cage (ANOVA, p < 0.01, post-hoc: HS lower than both MS and NS (p < 0.01)) (figure 6A). On the contrary, the mean PPA values in the HS group were the highest among all three groups (ANOVA: second last day, p < 0.05, post-hoc: HS significantly higher than MS (p < 0.05) but not NS; last day, p < 0.01, post-hoc: HS significantly higher than both MS and NS (p < 0.01) (figure 6B). NA was significantly higher in the HS group than the rest on the second last day (p < 0.01), but not significantly different on the last day (figure 6C); FA was significantly lower (p < 0.01) in the HS group than the MS group on the last day in the ABA cage (figure 6D).

**Figure 7:**
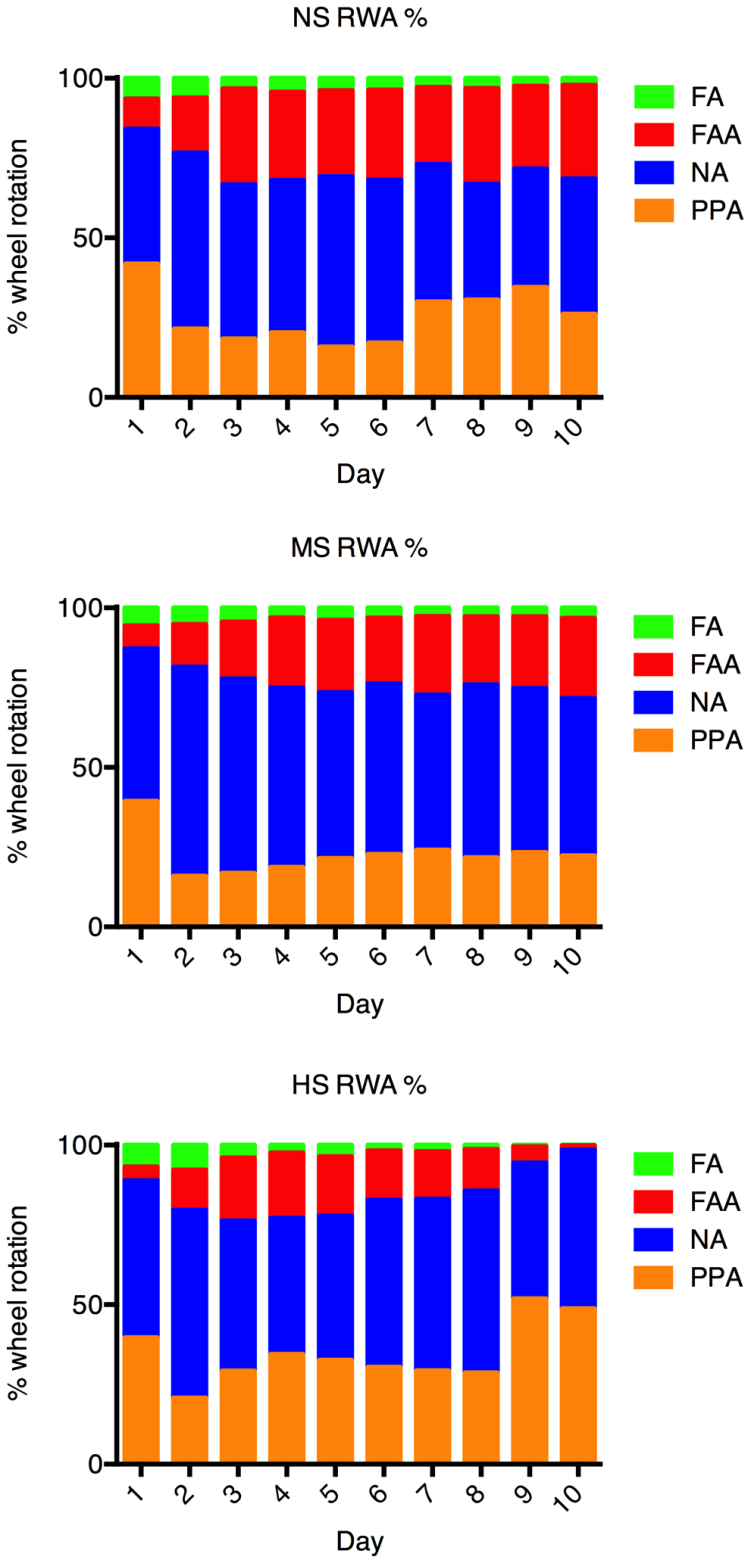
Composition of daily running wheel activities of different groups of rats. The percentage of FAA increased and stabilized at approximately one quarter of daily running wheel activities in the NS and the MS groups. Despite the decreasing trend of FAA percentage in the HS group (day 5: 19%, day 10: 1%), PPA was showing a clear increase, accounting for 49% of daily running wheel activity on day 10, nearly double of the PPA percentages in the NS (26%) and the MS groups (22%) on the same day.

Figure 7 illustrates the compositions of RWA across different groups. The percentage of FAA increased and stabilized in the NS (day 1: 9%, day 5: 27%, day 10: 29%) and the MS groups (day 1: 7%, day 5: 22%, day 10: 25%), but started to decrease in the HS group after day 5 (day 1: 4%, day 5: 19%, day 10: 1%). Percentage of PPA, on the other hand, was increasing in the HS group, constituting 49% of the total daily RWA on day 10 (26% and 22% in the NS and the MS groups, respectively).

## Discussion

To our knowledge, this paper is the first to relate percentage body weight loss and different RWA (FAA, PPA, NA and FA) in a relatively large cohort of 56 ABA rats. The original aim was to find a predictor among RWA during different periods, which may prognosticate percentage of body weight loss in advance. Total daily RWA was higher in the HS group, supporting the previously described correlation between body weight loss and hyperactivity^16^. We were expecting FAA, a behavioral phenomenon frequently used to evaluate hyperactivity in the ABA model, to be directly proportional to, and the most discriminating predictor of percentage body weight loss^16^. However, our results did not support this.

Though rats in the HS group were manifesting the most severe and rapid degree of percentage body weight loss, FAA in the HS group was not significantly higher than FAA in the other two groups throughout the entire ABA procedure. There was even a sharp drop of FAA in the HS group on the last two days. One may argue that this was caused by emaciation, but this cannot explain why PPA remains at a relatively high level among the same group of rats on the same days (figure 3 and 6). Similar to FAA, PPA was increasing over time in all rats undergoing restricted feeding, but unlike FAA, PPA in the HS group was increasing at a faster speed than those in the MS and the NS group, showing significantly higher RWA during this period of time than rats with less percentage body weight loss. Pearson correlations between total percentage body weight loss and average daily RWA during different periods showed a surprisingly negative correlation (−0.27, p < 0.05) between percentage body weight loss and FAA (figure 4). We believed this negative correlation was partially caused by bias (e.g. FAA decreased in rats with most body weight loss because of energy depletion, total percentage body weight loss versus average daily RWA of each individual rat was a rough estimation of the relation between weight loss and hyperactivity). Nonetheless, Pearson correlations between total percentage body weight loss and PPA and NA remained positive (0.49 and 0.35, respectively, p < 0.01 in both cases). ROC AUC analysis reconfirmed its superior predicting capacity on body weight loss over FAA. A drop in daily RWA occurred on day 9 in the HS group, and while FAA, NA, and FA were all decreasing, PPA was the only RWA component that was still elevated (figure 2 and 3). Figure 6 further refutes the possibility that this result was due to distortion of raw data during dropouts: PPA on the last day in the ABA cage (the day before dropout) in the HS group remained significantly higher than the other groups, but FAA was significantly lower in the HS group than the rest. These results challenge the present theory that decreased FAA implied symptom improvement in the rat ABA model.

**Figure 8:**
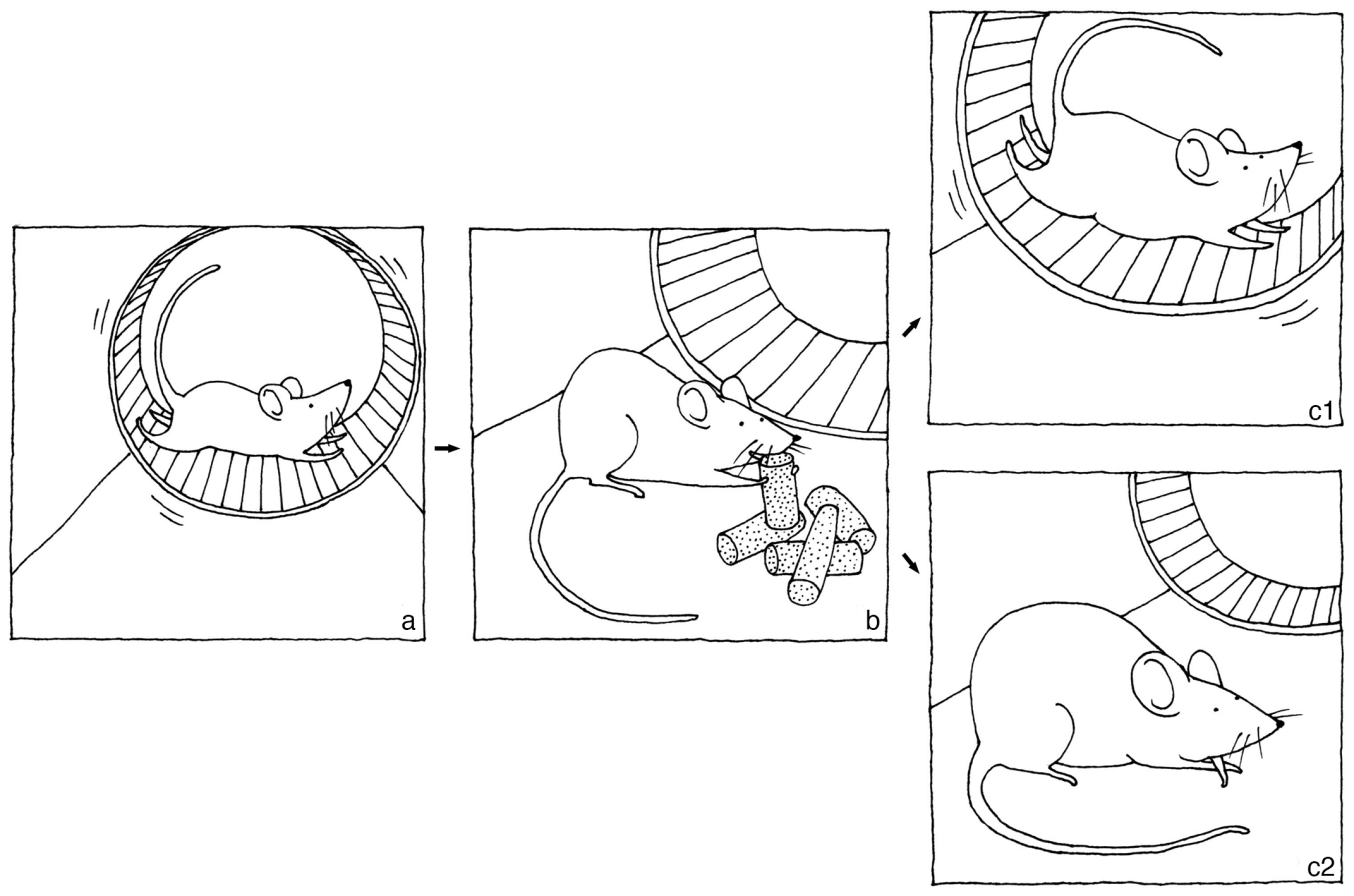
Change in body weight in relation to individual pre- and postprandial hyperactivity in rats in the activity-based anorexia (ABA) model. (a): Rats in the ABA model (scheduled feeding and access to running wheel) manifest hyperactivity 2–3 hours prior to feeding (food anticipatory activity). This is a general phenomenon. (b): Scheduled feeding. (c1): Rats with a tendency to run more after the feeding period (higher postprandial activity) are subjected to severe weight loss in the ABA model. (c2): Rats running less after the feeding period (lower postprandial activity) are less likely to lose a substantial amount of body weight. Drawing by Stephany Peiyen Hsiao.

We concluded the following based on our experimental results: 1) rats in the NS group (refractory, or able to maintain above 85% body weight, after 10 days in the ABA model) did not run significantly less than rats in the HS group (rats suffering from the most severe level of body weight loss) 2.5 hours before feeding (FAA); 2) rats in the HS group ran more than rats in the NS group during the 8-hour period after feeding (PPA); 3) body weight loss in rats in the ABA model was more directly proportional to raises in PPA, or failure to “rest” after scheduled feeding. If excessive RWA was related to body weight loss, we discovered that it was not FAA but PPA (or NA) that was playing a vital role in the weight loss in the ABA model; FAA was a more “common” behavioral phenomenon, exhibited by most rats under scheduled feeding, whereas PPA was a more “distinctive” feature, causing higher percentage weight loss if postprandial hyperactivity was manifested. Figure 8 summarizes our conclusions.

Numerous studies have been conducted to unravel the underlying mechanisms of FAA^16^. Leptin and ghrelin, for example, are hormones believed to have certain influences on FAA. While manipulations of these hormones in animal models proved their effects on FAA and RWA in general, PPA was excluded from assessment. Moreover, psychopharmacological studies aiming at relieving hyperactivity in the ABA model rarely take PPA into account.

Both insufficient food intake and increase in RWA contribute to drastic weight loss in the ABA model. Our observation that PPA, not FAA, is the key RWA reflecting body weight loss, contradicts previous theories. FAA increased in virtually all rats undergoing food restriction, and it remains a main feature of hyperactivity in this rodent model of anorexia nervosa. But whether it is the key factor leading to individual differences in terms of body weight loss, and whether decreased FAA indicates symptom alleviation in this animal model of anorexia nervosa, seems debatable at this moment. Our analysis on RWA data suggests PPA to be more positively correlated to percentage body weight loss than FAA. It may be worthwhile in future studies with the ABA model to include PPA and RWA during other periods in addition to FAA as a behavioral measure, during investigation of underlying mechanism and/or treatment of hyperactivity.

## Methods

56 female Wistar rats were included in our study. The body weight of each rat upon arrival was 200–250 g. All rats were housed individually on a 12:12 hour light:dark cycle (light onset = 07 AM) and ambient temperature was maintained at ±20 degrees Celsius. Rats were given one week of acclimatization (food and water ad libitum) in a standard home cage, prior to the start of the ABA procedure. The research projects were approved by the university ethics committee for laboratory experimentation (project numbers: 045/2006 and 046/2007), and were in accordance with the Belgian and European laws, guidelines and policies for animal experimentation, housing and care (Belgian Royal Decree of 29 May 2013 and European Directive 2010/63/EU on the protection of animals used for scientific purposes of 20 October 2010).

Starting on Day 0, each rat was introduced in/moved to an individual ABA cage (36 × 36 cm; custom-made) with a running wheel (35 cm in diameter, one rotation corresponds to a distance of approximately 110 cm; Campden Instruments, Loughborough, UK) at 11AM after baseline body weight was measured. Water was available ad libitum in the cage, but each rat was under food restriction: 50 g of food was introduced at 9:30AM and the remainders removed at 11AM, starting from Day 1 for a period of 10 consecutive days (1.5 hours of food access per day). Food intake and body weight of each rat were measured daily at 11AM after the feeding period ended. If body weight dropped below 70% of baseline, the rat would be removed from the model for ethics reasons and experiments ended prematurely in these rats. Running wheel activity (RWA) was monitored in LabView 7.0 (National Instruments, Austin, TX, USA) via position registration of an electro-magnetic rotary encoder (TWK-Elektronik GmbH, Dusseldorf, Germany) attached to the running wheel. After 10 consecutive days, all rats were removed from the ABA model.

The daily RWA was divided into 4 periods: FAA (2.5 hours, from 7:00AM to 9:30AM), feeding activity (FA, 1.5 hours, from 9:30AM to 11AM), postprandial activity (PPA, 8 hours, from 11AM to 7PM), and nocturnal activity (NA, 12 hours, from 7PM to 7AM next day).

Based on the percentage of baseline body weight at the end of the experiment, rats were categorized into three different groups: highly susceptible to ABA (HS, body weight reached below 70% of baseline within 10 days), moderately susceptible to ABA (MS, body weight between 70% and 85% of baseline after 10 days), and not susceptible to ABA (NS, body weight above 85% of baseline after 10 days) (85% and 70% of baseline body weight were predefined values based on previous studies)^13^,^14^,^15^.

An independent Mann-Whitney U Test (U test) or one-way analysis of variance (ANOVA) and post-hoc Tukey-Kramer test was performed to investigate the difference between groups. To explore the relationships between body weight loss and hyperactivity, Pearson correlation coefficients (total percentage body weight loss and average daily RWA) were calculated. To test how well RWA during different periods could distinguish between two diagnostic groups (non-responders (NS group) and responders (MS and HS groups)), the area under the receiver operating characteristic curve (ROC AUC) was calculated and compared. All statistical analyses were performed using Statistica (StatSoft, Oklahoma, U.S.A.), significance level p < 0.05.

## Author Contributions

H.W. drafted the manuscript, K.v.K. initiated the work of this manuscript, K.v.K. and L.L. collected the data, H.W., K.v.K. and T.T. analyzed the data, H.W., T.T. and L.L. contributed to statistical analysis and figures 1–7, and H.W., J.M.A. and B.N. finalized the submitted manuscript. All contributors critically reviewed and approved the manuscript.
